# Burden of musculoskeletal disorders in the gulf cooperation council countries, 1990–2019: Findings from the global burden of disease study 2019

**DOI:** 10.3389/fmed.2022.855414

**Published:** 2022-10-04

**Authors:** Hosam Alzahrani, Mansour A. Alshehri, Mazyad Alotaibi, Ahmed Alhowimel, Faris Alodaibi, Dalyah Alamam, Yan Zheng, Stefanos Tyrovolas

**Affiliations:** ^1^Department of Physical Therapy, College of Applied Medical Sciences, Taif University, Taif, Saudi Arabia; ^2^Physiotherapy Department, Faculty of Applied Medical Sciences, Umm Al-Qura University, Mecca, Saudi Arabia; ^3^NHMRC Centre of Clinical Research Excellence in Spinal Pain, Injury and Health, School of Health and Rehabilitation Sciences, University of Queensland, Brisbane, QLD, Australia; ^4^Department of Health and Rehabilitation Sciences, College of Applied Medical Sciences, Prince Sattam Bin Abdulaziz University, Al-Kharj, Saudi Arabia; ^5^Department of Health Rehabilitation Sciences, King Saud University, Riyadh, Saudi Arabia; ^6^WHO Collaborating Centre for Community Health Services (WHOCC), School of Nursing, The Hong Kong Polytechnic University, Hong Kong, Hong Kong SAR, China; ^7^Research, Innovation and Teaching Unit, Parc Sanitari Sant Joan de Déu, Fundacio Sant Joan de Deu, Sant Boi de Llobregat, Spain; ^8^Instituto de Salud Carlos III, Centro de Investigación Biomédica en Red de Salud Mental (CIBERSAM), Madrid, Spain

**Keywords:** global burden of disease, Gulf Cooperation Council (GCC), musculoskeletal – disorders, prevalence, years lived with disability

## Abstract

**Objective:**

The purpose of this study was to investigate the burden of musculoskeletal (MSK) health conditions in Gulf Cooperation Council (GCC) countries based on the Global Burden of Disease (GBD) data.

**Methods:**

The data for GCC countries were obtained from the 2019 GBD study to evaluate the burden of MSK disorders which include the following countries: Bahrain, Kuwait, Oman, Qatar, Saudi Arabia, and the United Arab Emirates (UAE). The main outcome measures were age-standardized prevalence and years of life lived with disability (YLDs) associated with MSK disorders. The burden of MSK disorders attributable to the category of behavioral, metabolic, or environmental/occupational was reported to estimate the risk-attributable fractions of disease.

**Results:**

MSK disorders prevalence ranked fifth in Kuwait, sixth in Bahrain, Oman, Qatar, and UAE, and seventh in Saudi Arabia among all the diseases in 2019. For all GCC countries, MSK disorders were ranked the second leading cause of disability as measured by YLDs for the years 1990 and 2019. The age-standardized prevalence of MSK disorders in 2019 for Bahrain, Kuwait, Oman, Qatar, Saudi Arabia, and UAE was 18.56% (95% UI: 17.51–19.66), 19.35% (18.25–20.52), 18.23% (17.14–19.36), 18.93% (17.81–20.06), 19.05% (17.96–20.22), and 18.26% (17.18–19.38), respectively. The age-standardized YLDs per 100,000 individuals of MSK disorders in 2019 for Bahrain, Kuwait, Oman, Qatar, Saudi Arabia, and UAE were 1,734 (1,250–2,285), 1,764 (1,272–2,322), 1,710 (1,224–2,256), 1,721 (1,246–2,274), 1,715 (1,230–2,274), and 1,681 (1,207–2,235), respectively. For risk factors, high body mass index (BMI) had the highest contribution to MSK disorders YLDs in most GCC countries (Bahrain, Kuwait, Oman, and Saudi Arabia), followed by the exposure to occupational ergonomic factors which had the highest contribution to MSK disorders YLDs in Qatar and UAE.

**Conclusion:**

There was an increase in both age-standardized prevalence of MSK disorders and YLDs between 1990 and 2019 that was observed for all GCC countries. Some risk factors such as higher BMI and exposure to occupational ergonomic factors were highly associated with YLDs due to MSK disorders. The results of this study provide guidance for the potential nature of preventative and management programs to optimize the individual’s health.

## Introduction

In the last century, communicable diseases were the most problematic health issue. However, with modern medical advancements and vaccination, the issue of infectious diseases became less of a problem with less mortality rate and an increase in life expectancy (1). Currently, non-communicable diseases (NCDs) that are related to lifestyle, including cardiovascular diseases, diabetes mellitus, and musculoskeletal (MSK) disorders, pose a new global threat to human health (2). In particular, from 1990 to 2017, NCDs became the leading cause of death (3). Furthermore, 80% of the disability in 2017 was driven by NCDs (4). In 2010, MSK disorders were one of the main contributors to global years lived with disability (YLDs) (5). The MSK disorders are expected to keep growing, with low back pain (LBP), neck pain, and osteoarthritis as the most prevalent conditions (6). The MSK conditions are highly prevalent and disabling (7). However, detailed data from different countries are still needed to compare results and allocate appropriate efforts to address the associated disability (8).

In countries of the Gulf Cooperation Council (GCC), for example, previous findings have shown a rising burden of NCDs such as LBP and neck pain (9). Neck pain and LBP were ranked in second place and became the leading cause of disability-adjusted life-years (DALYs) in 2013 in Bahrain, Oman, Qatar, and Saudi Arabia (9). Consistently, LBP ranked second over the period 1990–2017 in Saudi Arabia (10). Furthermore, in Saudi Arabia, YLDs due to metabolic, behavioral, and environmental or occupational risk factors [e.g., high body mass index (BMI)] have been rising across all age groups, which increase the burden of MSK disorders (10). The Gulf countries have made enormous advances in their health systems in a short time; however, many lifestyle-related risk factors, such as obesity, diabetes, and metabolic syndrome (11, 12) were highly reported, leading to an increasing burden of NCDs (10).

Recently, life in the GCC has changed significantly, resulting in epidemiological transitions, such as obesity, diabetes, and other chronic diseases were growing to be the leading causes of disease and death rates (13). This rising disease pattern is often attributed to behavioral and environmental risk factors such as physically inactive lifestyles (13, 14). As such, one key research area prioritized by researchers in the region was the need to investigate the burden and experience of GCC residents with MSK health conditions, and to explore the associated disability. In this current research, we presented the prevalence of MSK disorders and YLDs from 1990 to 2019, as well as the attributable burden from the risk factors of MSK disorders. This study aimed to report findings on MSK disorders between 1990 and 2019 from the 2019 global burden of diseases (GBD) in the GCC countries. This research represents an important step in the region’s Cooperation Council goals because related risk factors are prevalent, especially physical inactivity and obesity, which are particularly high (15). Understanding the burden of MSK health conditions in a Gulf context will assist researchers to compare the results with other countries and policymakers to allocate efforts and develop relevant best-practice guidelines for the prevention and management of MSK disorders.

## Materials and methods

### Overview

All the data and analyses used in this study were obtained from the GBD study 2019, which is publicly available online on the website of the Institute of Health Metrics and Evaluation.^1^ Briefly, the GBD 2019 covers 204 countries and territories grouped into 21 regions and seven super-regions from 1990 to 2019 for 369 diseases and injuries. Full descriptions of the methodological approaches used generally for the GBD 2019 and specifically for MSK have been provided elsewhere (7, 16).

We evaluated the burden of MSK disorders in the GCC, which contains six countries: Bahrain, Kuwait, Oman, Qatar, Saudi Arabia, and the United Arab Emirates (UAE). The GCC is a regional cooperation system among the Arab countries of the Gulf. The geographical proximity of the GCC countries and the similarity of their regulations, customs and traditions, and economic and social conditions were among the factors that these countries share.^2^

### Case definition

The International Classification of Diseases (ICD) codes assigned for MSK disorders in the GBD are listed in Supplementary Appendix Table 1. In summary, the list of MSK disorders included in this study were rheumatoid arthritis, osteoarthritis, osteoarthritis hip, osteoarthritis knee, osteoarthritis hand, osteoarthritis other, LBP, neck pain, gout, and other MSK disorders (17).

### Input data

For the process of estimation of diseases or injuries, the GBD relies on the identification of various relevant data sources. These data sources include, for example, household surveys, censuses, civil registration and vital statistics, health service use, disease surveillance systems, and other sources. Each type of these data was identified through a systematic review of published studies, searches of international and government organization websites, published reports, primary data sources such as national health surveys, and contributions of datasets by collaborators of GBD.

### Outcome measures

The main outcome measures of this study were the age-standardized prevalence and YLDs associated with MSK disorders. The prevalence of a specific condition refers to “the proportion of individuals who have that condition at a particular time”. YLDs refer to “years of healthy life lost as a result of any sort of health-related disability over time”, and are calculated as the product of the disability weight and the prevalence estimate. The disability weight is a value between 0 (full health) and 1 (death) which represents the health loss severity associated with a particular disease. A detailed description of the disability weights used in the GBD 2019 has been reported elsewhere (16).

The DisMod-MR 2.1, a Bayesian meta-regression tool, was used for generating the estimates of prevalence (18). Model-based prevalence estimates, in combination with disability weights, were used to calculate the cause-specific YLDs for each age, sex, location, and year.

The 95% uncertainty intervals (95% UI) for each estimate, including prevalence and YLDs, were calculated on the basis of 1,000 draws at each step of the estimation process of the computation, bounded by the 2.5th and 97.5th percentiles in the distribution. More details on the calculation of uncertainty intervals are reported elsewhere (19).

### Risk estimation

The burden (the metric being assessed here was YLDs) of MSK disorders attributable to the category of behavioral, metabolic, or environmental/occupational was reported to estimate the risk-attributable fractions of disease (20). The specific risk factors that were included in GBD 2019 and were considered to have enough data to establish a causal association with MSK disorders were: occupational ergonomic factors, high BMI, kidney dysfunction, and smoking (20). The relative risks were extracted from published meta-analyses (20).

## Results

### Prevalence

Globally, MSK disorders were ranked as one of the top 10 prevalent conditions for the years 1990 and 2019 (Table 1). In 2019, MSK disorders ranked fifth in Kuwait, sixth in Bahrain, Oman, Qatar, and UAE, and seventh in Saudi Arabia among all the diseases studied.

**TABLE 1:** Age-standardized prevalence and YLDs rankings of MSK disorders in GCC countries in 1990 and 2019 (out of all causes).

Country	Prevalence		YLDs ranking	
	1990	2019	1990	2019
Global	9	7	1	1
Bahrain	7	6	2	2
Kuwait	6	5	2	2
Oman	9	6	2	2
Qatar	8	6	2	2
Saudi Arabia	8	7	2	2
UAE	7	6	2	2

GCC, Gulf Cooperation Council; MSK, musculoskeletal; YLDs, years lived with disability.

**FIGURE 1:**
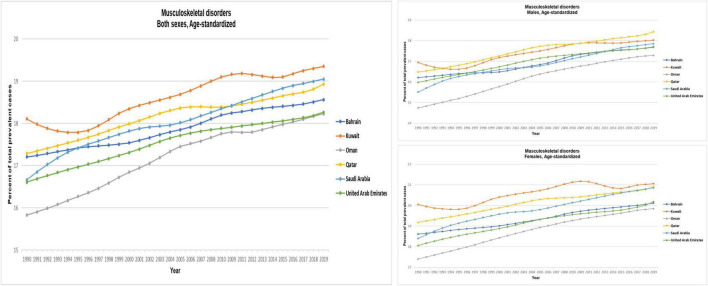
Age-standardized prevalence (%) of MSK disorders for GCC countries, Global Burden of Disease 2019 study. The figure is adopted by the Institute for Health Metrics and Evaluation [IHME] (21).

The age-standardized estimate of MSK disorders globally and in the GCC countries, by gender, for 1990 and 2019 is presented in Figure 1 and Table 2. Globally, the age-standardized prevalence of MSK disorders in 1990 was 18.88% (95% UI: 17.87–19.92), and this increased to 19.21% (18.20–20.27) in 2019. The prevalence was higher in women than men. The prevalence in women was 21.09% (19.99–22.21) in 1990 and 21.24% (20.16–22.39) in 2019; whereas for men, the prevalence was 16.51% (15.56–17.49) in 1990 and 17.02% (16.09–18.02) in 2019.

**TABLE 2:** Age-standardized prevalence (%) and YLDs (rates per 100,000 population) of MSK disorders in 1990 and 2019, and the corresponding percentage change between 1990 and 2019, in GCC countries.

		Age-standardized prevalence					Age-standardized YLDs (rates per 100,000 population)				
		1990		2019		% Change	1990		2019		% Change
Country	Sex	Value	95% UI	Value	95% UI		Value	95% UI	Value	95% UI	
Global	Total	18.88	17.87–19.92	19.21	18.20–20.27	1.75 (0.82–2.66)	1,817	1,303–2,404	1,791	1,288–2,367	1.56 (−0.19–3.30)
	Men	16.51	15.56–17.49	17.02	16.09–18.02	3.11 (2.13–4.12)	1,517	1,086–2,016	1,505	1,078–1,995	2.41 (0.47–4.36)
	Women	21.09	19.99–22.21	21.24	20.16–22.39	0.71 (−0.22–1.69)	2,103	1,514–2,771	2,064	1,487–2,728	1.08 (−0.64–2.73)
Bahrain	Total	17.21	16.15–18.29	18.56	17.51–19.66	7.88 (5.49–10.25)	1,619	1,162–2,153	1,734	1,250–2,285	7.31 (3.29–11.66)
	Men	16.22	15.16–17.34	17.70	16.67–18.80	9.15 (5.98–12.24)	1,501	1,073–1,994	1,616	1,158–2,146	6.38 (1.25–11.77)
	Women	18.61	17.45–19.76	20.12	18.93–21.34	8.10 (5.34–11.00)	1,781	1,284–2,349	1,930	1,32–2,535	9.56 (5.05–14.36)
Kuwait	Total	18.11	17.17–19.10)	19.35	18.25–20.52	6.86 (3.55–10.62)	1,662	1,198–2,181	1,764	1,272–2,322	3.11 (−0.98–7.18)
	Men	16.94	15.99–17.96	18.03	16.92–19.14	6.40 (2.38–10.57)	1,519	1,092–2,014	1,596	1,154–2,124	2.29 (−2.44–7.39)
	Women	20.05	19.01–21.16	21.06	19.83–22.34	5.06 (1.50–9.26)	1,892	1,361–2,482	1,985	1,432–2,613	5.65 (0.89–10.42)
Qatar	Total	17.29	16.19–18.40	18.93	17.81–20.06	9.50 (7.01–12.17)	1,510	1,076–2,002	1,721	1,246–2,274	8.59 (4.81–12.98)
	Men	16.49	15.36–17.59	18.43	17.29–19.60	11.80 (8.51–15.32)	1,394	985–1,851	1,641	1,180–2,168	9.32 (4.37–14.73)
	Women	19.18	18.00–20.42	20.90	19.71–22.16	8.97 (6.25–11.88)	1,675	1,203–2,215	1,971	1,430–2,578	7.69 (3.61–12.02)
Oman	Total	15.83	14.77–16.89	18.23	17.14–19.36	15.16 (12.35–17.85)	1,583	1,138–2,092	1,710	1,224–2,256	15.44 (11.10–19.81)
	Men	14.74	13.67–15.79	17.29	16.21–18.43	17.26 (13.54–21.29)	1,477	1,049–1,958	1,591	1,138–2,118	14.81 (9.12–20.45)
	Women	17.40	16.26–18.52	19.84	18.67–21.18	14.01 (10.73–17.48)	1,806	1,301–2,376	1,907	1,369–2,512	16.10 (10.86–21.47)
Saudi Arabia	Total	16.66	15.63–17.79	19.05	17.96–20.22	14.33 (11.90–17.00)	1,500	1,068–1,996	1,715	1230–2274	12.04 (8.09–16.41)
	Men	15.51	14.47–16.65	17.85	16.74–19.00	15.12 (11.73–18.75)	1,376	968–1,838	1,565	1,116–2,079	10.36 (5.39–15.73)
	Women	18.40	17.26–19.65	20.86	19.66–22.15	13.36 (10.38–16.52)	1,686	1,191–2,234	1,942	1,396–2,562	14.43 (9.60–19.56)
UAE	Total	16.60	15.58–17.60	18.26	17.18–19.38	9.99 (7.48–12.41)	1,541	1,108–2,044	1,681	1,207–2,235	7.45 (3.27–11.64)
	Men	15.98	14.99–16.98	17.68	16.57–18.77	10.63 (7.39–14.04)	1,473	1,055–1,961	1,602	1,153–2,111	5.98 (0.94–11.11)
	Women	18.06	16.98–19.21	20.18	18.95–21.53	11.75 (8.83–14.96)	1,687	1,214–2,232	1,902	1,360–2,511	11.81 (7.31–16.37)

GCC, Gulf Cooperation Council; MSK, musculoskeletal; UI, uncertainty interval; YLDs, years lived with disability; % Change, percentage change between 1990 and 2019.

Of the six GCC countries, the highest age-standardized prevalence of MSK disorders reported in 2019 was in Kuwait [19.35% (18.25–20.52)], followed by Saudi Arabia [19.05% (17.96–20.22)] (Table 2). The lowest prevalence was in Oman [18.23% (17.14–19.36)] and UAE [18.26% (17.18–19.38)]. There was an increase in the age-standardized prevalence between 1990 and 2019 that was observed for all GCC countries, with Oman reporting the largest increase [15.16% (12.35–17.85)] and Kuwait reporting the smallest increase [6.86% (3.55–10.62)].

Globally, LBP was the highest prevalent out of all MSK disorders in 2019 (Table 3). The global age-standardized prevalence of LBP was 7.28% (6.47–8.18). Of the six GCC countries, the highest age-standardized prevalence of LBP in 2019 was observed in Qatar [7.98% (7.00–9.04)], followed by Kuwait [7.94% (6.99–8.94)].

**TABLE 3:** Age-standardized prevalence (%) for different MSK disorders in the GCC countries in 2019 and the percentage change between 1990 and 2019.

		Global		Bahrain		Kuwait		Qatar		Oman		Saudi Arabia		UAE	
Cause	Sex	Prevalence (95% UI)	% Change	Prevalence (95% UI)	% Change	Prevalence (95% UI)	% Change	Prevalence (95% UI)	% Change	Prevalence (95% UI)	% Change	Prevalence (95% UI)	% Change	Prevalence (95% UI)	% Change
Rheumatoid arthritis	Total	0.23 (0.21–0.26)	9.25 (8.66–9.83)	0.16 (0.14–0.19)	37.20 (29.33–45.04)	0.12 (0.10–0.14)	34.91 (27.78–41.86)	0.11 (0.09–0.13)	32.82 (26.05–39.61)	0.10 (0.08–0.12)	40.10 (33.84–46.53)	0.10 (0.09–0.12)	45.42 (39.22–51.91)	0.10 (0.08–0.11)	25.28 (19.78–31.68)
	Men	0.14 (0.13–0.16)	11.57 (10.87–12.27)	0.08 (0.07–0.10)	41.35 (32.25–51.23)	0.08 (0.07–0.10)	26.40 (18.95–34.35)	0.08 (0.07–0.10)	36.08 (27.83–44.91)	0.07 (0.06–0.08)	44.23 (36.34–53.24)	0.07 (0.06–0.09)	45.12 (36.50–53.28)	0.07 (0.06–0.08)	30.66 (23.10–38.82)
	Women	0.32 (0.29–0.35)	8.52 (7.85–9.17)	0.30 (0.26–0.35)	48.08 (37.08–60.35)	0.17 (0.15–0.20)	27.28 (18.33–35.55)	0.20 (0.17–0.24)	45.56 (34.12–57.84)	0.16 (0.13–0.18)	42.89 (33.50–52.67)	0.15 (0.13–0.18)	44.03 (34.62–53.81)	0.17 (0.14–0.20)	30.34 (21.23–40.48)
Osteoarthritis	Total	6.63 (6.02–7.33)	3.94 (2.79–5.07)	5.71 (5.12–6.31)	7.57 (4.66–10.82)	6.02 (5.42–6.68)	9.78 (6.64–13.14)	5.87 (5.28–6.54)	5.97 (2.13–9.45)	5.59 (5.04–6.20)	16.88 (13.50–20.38)	6.92 (6.22–7.72)	11.14 (8.24–14.00)	5.59 (5.03–6.18)	9.68 (6.08–13.26)
	Men	5.62 (5.10–6.21)	5.09 (4.03–6.06)	5.32 (4.76–5.92)	9.52 (5.12–14.49)	5.59 (4.98–6.23)	9.34 (4.21–14.02)	5.66 (5.07–6.31)	8.71 (3.18–13.42)	5.14 (4.59–5.70)	19.32 (14.33–24.61)	6.45 (5.79–7.19)	12.47 (8.35–16.87)	5.33 (4.78–5.92)	12.44 (7.32–17.69)
	Women	7.51 (6.83–8.29)	3.51 (2.21–4.76)	6.35 (5.68–7.03)	8.63 (4.44–12.91)	6.67 (5.97–7.40)	8.89 (4.38–13.53)	6.67 (5.99–7.40)	8.08 (4.27–12.63)	6.19 (5.57–6.90)	16.12 (11.72–20.97)	7.62 (6.81–8.50)	10.25 (6.79–14.09)	6.39 (5.73–7.05)	11.23 (6.62–15.88)
Osteoarthritis hip	Total	0.42 (0.33–0.52)	9.85 (8.53–11.48)	0.34 (0.26–0.43)	22.50 (17.08–29.32)	0.37 (0.29–0.47)	22.37 (16.18–28.65)	0.40 (0.30–0.50)	20.57 (13.73–27.74)	0.31 (0.24–0.39)	50.10 (42.14–58.80)	0.35 (0.27–0.44)	38.92 (31.07–46.14)	0.35 (0.27–0.44)	32.43 (24.33–39.95)
	Men	0.42 (0.32–0.52)	12.37 (11.18–13.78)	0.36 (0.28–0.46)	20.69 (12.18–30.59)	0.40 (0.30–0.50)	21.47 (12.44–29.96)	0.41 (0.32–0.52)	17.91 (9.27–26.45)	0.33 (0.25–0.41)	48.19 (39.06–59.71)	0.37 (0.28–0.46)	37.57 (27.26–46.81)	0.36 (0.28–0.46)	30.47 (19.68–40.26)
	Women	0.42 (0.33–0.52)	8.31 (6.58–10.31)	0.31 (0.24–0.39)	23.13 (15.51–32.62)	0.34 (0.26–0.44)	25.57 (17.71–34.17)	0.35 (0.27–0.45)	21.12 (12.09–31.71)	0.29 (0.22–0.36)	51.36 (39.60–62.99)	0.32 (0.25–0.40)	40.72 (31.48–51.39)	0.31 (0.24–0.39)	32.89 (23.62–42.10)
Osteoarthritis knee	Total	4.57 (3.96–5.21)	8.66 (7.99–9.30)	4.17 (3.56–4.80)	10.02 (5.69–14.96)	4.42 (3.79–5.08)	12.36 (7.40–17.59)	4.30 (3.68–4.97)	7.77 (2.50–12.94)	4.06 (3.49–4.69)	21.17 (16.19–26.14)	4.21 (3.58–4.84)	17.64 (12.20–22.61)	4.09 (3.49–4.74)	12.49 (7.33–17.54)
	Men	3.71 (3.20–4.26)	8.52 (7.66–9.23)	3.86 (3.29–4.47)	11.54 (4.88–19.63)	4.06 (3.49–4.69)	11.45 (3.71–18.55)	4.12 (3.52–4.78)	10.40 (2.15–17.72)	3.71 (3.20–4.30)	22.43 (15.42–30.46)	3.88 (3.30–4.49)	18.41 (10.87–26.74)	3.88 (3.32–4.54)	14.84 (6.77–22.07)
	Women	5.32 (4.60–6.07)	9.16 (8.39–9.94)	4.67 (3.96–5.42)	11.68 (5.20–18.22)	4.94 (4.20–5.69)	11.91 (5.26–19.16)	4.95 (4.24–5.73)	10.65 (4.18–17.72)	4.52 (3.87–5.26)	21.59 (14.59–28.97)	4.72 (4.03–5.46)	17.53 (10.45–25.57)	4.71 (4.04–5.44)	15.07 (7.73–22.38)
Osteoarthritis hand	Total	1.80 (1.38–2.36)	−7.54 (−9.99– −5.15)	0.88 (0.65–1.19)	−4.46 (–5.37– −3.64)	0.92 (0.68–1.24)	2.36 (1.33–3.51)	0.78 (0.57–1.06)	−10.30 (−12.53– −8.41)	0.91 (0.67–1.23)	−1.15 (−1.82– −0.44)	2.33 (1.74–3.08)	0.49 (−0.06–1.04)	0.80 (0.59–1.08)	−7.65 (−9.27– −6.19)
	Men	1.28 (0.98–1.68)	−6.54 (−8.89– −4.13)	0.60 (0.43–0.82)	1.19 (0.62–1.78)	0.61 (0.44–0.84)	0.84 (−0.13–1.88)	0.61 (0.44–0.84)	1.33 (0.64–2.02)	0.59 (0.43–0.81)	2.67 (1.93–3.51)	1.95 (1.44–2.62)	1.89 (1.25–2.58)	0.60 (0.43–0.82)	1.15 (0.52–1.81)
	Women	2.25 (1.71–2.93)	−7.44 (−10.11– −4.74)	1.32 (0.97–1.76)	0.44 (0.20–0.70)	1.35 (1.00–1.81)	0.36 (−0.31–1.02)	1.33 (0.99–1.79)	0.56 (0.22–0.90)	1.32 (0.97–1.76)	1.13 (0.77–1.55)	2.90 (2.15–3.81)	0.59 (0.18–0.99)	1.33 (0.98–1.78)	0.46 (0.07–0.86)
Osteoarthritis other	Total	0.78 (0.60–0.99)	6.51 (5.47–7.41)	0.92 (0.69–1.18)	9.48 (4.76–14.47)	0.96 (0.72–1.25)	6.96 (1.99–12.03)	1.01 (0.75–1.30)	9.73 (4.47–15.32)	0.88 (0.66–1.13)	19.68 (14.12–25.96)	0.93 (0.69–1.19)	14.21 (9.64–19.83)	0.93 (0.69–1.20)	12.91 (8.00–18.91)
	Men	0.84 (0.64–1.07)	8.11 (6.98–9.15)	1.00 (0.74–1.28)	8.09 (2.14–15.05)	1.05 (0.78–1.35)	6.98 (0.25–13.81)	1.07 (0.79–1.38)	7.25 (0.86–14.11)	0.96 (0.72–1.24)	18.85 (11.99–27.25)	1.01 (0.74–1.31)	14.00 (7.83–21.72)	0.98 (0.73–1.28)	10.76 (5.23–19.22)
	Women	0.72 (0.55–0.90)	4.73 (3.60–5.73)	0.80 (0.61–1.03)	8.51 (2.71–15.52)	0.85 (0.64–1.08)	9.16 (2.21–16.19)	0.85 (0.64–1.10)	8.18 (1.64–14.89)	0.78 (0.58–1.00)	18.92 (11.35–26.44)	0.81 (0.61–1.04)	14.11 (6.35–21.03)	0.80 (0.60–1.02)	12.32 (5.16–19.07)
Low back pain	Total	7.28 (6.47–8.18)	−15.51 (−16.29– −14.69)	7.53 (6.62–8.51)	−0.16 (−4.01–4.09)	7.94 (6.99–8.94)	0.85 (−3.04–4.97)	7.98 (7.00–9.04)	1.20 (−2.78–5.86)	7.51 (6.60–8.55)	1.05 (−2.93–5.50)	7.51 (6.58–8.50)	1.65 (−2.02–5.45)	7.47 (6.55–8.44)	0.36 (−4.06–5.15)
	Men	6.27 (5.58–7.04)	−14.96 (−15.71– −14.15)	7.74 (6.82–8.78)	−0.02 (−5.55–5.80)	7.88 (6.93–8.89)	1.67 (−3.50–7.08)	8.15 (7.14–9.26)	1.26 (−4.01–6.94)	7.68 (6.74–8.77)	1.54 (−3.62–7.48)	7.74 (6.78–8.79)	2.17 (−3.14–7.77)	7.68 (6.70–8.70)	−1.15 (−6.24–4.71)
	Women	8.20 (7.27–9.24)	−15.97 (−16.93– −15.04)	7.17 (6.27–8.10)	−0.23 (−5.71–5.66)	8.04 (7.08–9.10)	0.44 (−5.04–6.54)	7.47 (6.53–8.46)	0.92 (−4.65–6.73)	7.12 (6.26–8.11)	0.05 (−5.27–5.69)	7.18 (6.30–8.21)	1.34 (−4.10–6.83)	6.93 (6.07–7.84)	3.74 (−2.18–10.03)
Neck pain	Total	2.81 (2.28–3.53)	0.59 (−1.59–2.77)	2.80 (2.21–3.55)	−1.22 (−2.08– −0.43)	2.32 (1.82–2.98)	3.79 (2.57–5.01)	2.68 (2.12–3.38)	−1.74 (−2.76– −0.74)	2.79 (2.21–3.53)	0.44 (−0.19–1.11)	2.86 (2.25–3.63)	1.45 (0.91–2.01)	2.67 (2.11–3.36)	−1.43 (−2.48– −0.48)
	Men	2.48 (2.01–3.11)	0.78 (−1.85–3.42)	2.34 (1.85–2.98)	1.19 (0.62–1.78)	1.91 (1.49–2.46)	0.85 (−0.13–1.88)	2.39 (1.89–3.02)	1.33 (0.64–2.02)	2.33 (1.83–2.96)	2.67 (1.93–3.51)	2.34 (1.84–2.97)	1.89 (1.25–2.58)	2.35 (1.85–2.98)	1.14 (0.52–1.81)
	Women	3.13 (2.51–3.94)	0.34 (−2.48–3.15)	3.62 (2.82–4.63)	0.44 (0.20–0.70)	2.84 (2.21–3.65)	0.37 (−0.31–1.02)	3.68 (2.85–4.70)	0.56 (0.22–0.90)	3.62 (2.81–4.63)	1.13 (0.77–1.55)	3.64 (2.83–4.66)	0.58 (0.18–0.99)	3.65 (2.83–4.68)	0.46 (0.07–0.86)
Gout	Total	0.68 (0.55–0.83)	23.69 (21.65–26.25)	0.64 (0.51–0.79)	15.25 (8.16–22.89)	0.66 (0.52–0.82)	7.03 (−0.14–15.73)	0.78 (0.62–0.98)	19.38 (11.09–28.67)	0.60 (0.47–0.75)	29.65 (21.91–38.06)	0.64 (0.50–0.79)	19.90 (12.58–27.09)	0.70 (0.55–0.87)	21.28 (12.78–30.47)
	Men	1.09 (0.88–1.33)	23.13 (21.06–25.66)	0.88 (0.70–1.09)	9.41 (1.87–17.99)	0.92 (0.74–1.15)	10.00 (2.03–20.26)	0.95 (0.75–1.19)	8.12 (0.06–17.51)	0.84 (0.66–1.06)	25.40 (16.38–35.14)	0.89 (0.70–1.12)	18.42 (10.11–26.66)	0.86 (0.68–1.08)	13.09 (4.28–23.13)
	Women	0.31 (0.25–0.39)	21.93 (19.82–24.34)	0.27 (0.21–0.34)	11.46 (3.37–20.17)	0.29 (0.23–0.37)	12.54 (4.39–20.96)	0.29 (0.23–0.36)	10.07 (2.84–18.15)	0.26 (0.20–0.33)	25.80 (15.53–35.72)	0.27 (0.21–0.34)	19.80 (10.76–29.10)	0.27 (0.21–0.34)	15.66 (7.79–24.86)
Other MSK disorders	Total	5.72 (4.93–6.64)	33.36 (29.92–37.62)	5.34 (4.58–6.24)	36.24 (26.84–45.50)	6.06 (5.16–7.11)	19.91 (6.36–34.99)	5.14 (4.29–6.09)	50.76 (39.01–64.30)	5.09 (4.26–5.99)	79.26 (63.76–97.86)	4.67 (3.83–5.61)	100.55 (78.98–129.32)	5.13 (4.28–6.08)	47.40 (36.22–60.51)
	Men	4.45 (3.76–5.24)	41.54 (36.37–48.39)	4.51 (3.81–5.33)	47.62 (32.79–64.43)	4.81 (4.00–5.76)	19.25 (2.46–38.20)	4.54 (3.73–5.45)	78.98 (58.32–107.16)	4.21 (3.44–5.10)	111.61 (82.83–153.41)	3.44 (2.67–4.31)	153.32 (103.82–236.06)	4.48 (3.66–5.37)	58.32 (41.20–78.70)
	Women	6.92 (6.04–7.93)	28.58 (25.79–31.99)	6.75 (5.84–7.84)	30.74 (21.64–41.30)	7.63 (6.58–8.92)	13.22 (1.22–27.91)	7.22 (6.21–8.38)	34.52 (23.90–45.27)	6.65 (5.63–7.74)	60.14 (45.65–76.58)	6.50 (5.51–7.62)	70.40 (52.27–91.10)	7.04 (5.99–8.32)	41.77 (30.65–54.81)

GCC, Gulf Cooperation Council; MSK, musculoskeletal; UI, uncertainty interval; YLDs, years lived with disability; % Change, percentage change between 1990 and 2019.

The specific MSK disorders that have shown the largest increases in the age-standardized prevalence among all the GCC countries between 1990 and 2019 were rheumatoid arthritis and osteoarthritis hip (Table 3). The largest increase in age-standardized prevalence of rheumatoid arthritis was observed in Saudi Arabia [45.42% (39.22–51.91)], and the smallest increase was observed in UAE [25.28% (19.78–31.68)]. The largest increase in age-standardized prevalence of osteoarthritis hip was observed in Oman [50.10% (42.14–58.80)], and the smallest increase was observed in Qatar [20.57 (13.73–27.74)].

### Years lived with disability

**FIGURE 2:**
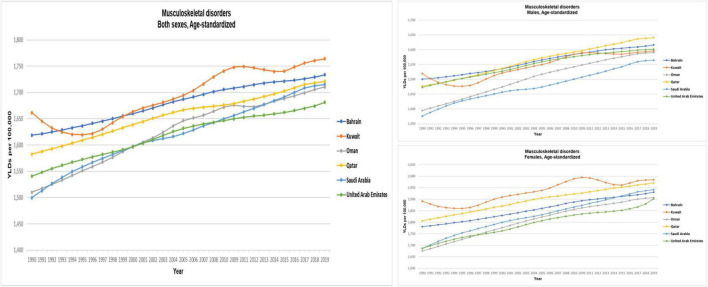
Age-standardized years lived with disability (rates per 100,000) from MSK disorders for GCC countries, both sexes, Global Burden of Disease 2019 study. The figure is adopted by the Institute for Health Metrics and Evaluation [IHME] (21).

Globally, MSK disorders were the leading cause of disability as measured by YLDs for the years 1990 and 2019 (Table 1 and Figure 2). For all six GCC countries, MSK disorders were ranked the second leading cause of disability as measured by YLDs for the years 1990 and 2019.

The age-standardized YLDs per 100,000 individuals of MSK disorders globally and in the GCC countries, by gender, for 1990 and 2019 are presented in Table 2. Globally, the age-standardized YLDs per 100,000 individuals of MSK disorders in 1990 was 1,817 (95% UI: 1,303–2,404), and this decreased to 1,791 (1,288–2,367) in 2019. The age-standardized YLDs per 100,000 individuals were higher in women than in men. For women, the age-standardized YLDs per 100,000 individuals in 1990 were 2,103 (1,514–2,771) and 2,064 (1,487–2,728) in 2019, while for men, it was 1,517 (1,086–2,016) in 1990 and 1,505 (1,078–1,995) in 2019.

Of the six GCC countries, the highest age-standardized YLDs per 100,000 individuals of MSK disorders 2019 were observed in Kuwait [1,764 (1,272–2,322)], followed by Bahrain [1,734 (1,250–2,285)] (Table 2). The lowest age-standardized YLDs per 100,000 individuals were observed in UAE [1,681 (1,207–2,235)], followed by Oman [1,710 (1,224–2,256)]. There was an increase in the age-standardized YLDs between 1990 and 2019 that was observed for all GCC countries, with Oman reporting the largest increase [15.44% (11.10–19.81)] and Kuwait reporting the smallest increase [3.11% (−0.98–7.18)].

Globally, LBP was the leading cause of YLDs out of all MSK disorders in 2019 (Table 4). The global age-standardized YLDs per 100,000 individuals for LBP were 780 (549–1,046). Of the six GCC countries, the highest age-standardized YLDs per 100,000 individuals for LBP were observed in Qatar [840 (592–1,133)], followed by Kuwait [836 (589–1,121)].

**TABLE 4:** Age-standardized YLDs (rates per 100,000 population) for different MSK disorders in the GCC countries in 2019 and the percentage change between 1990 and 2019.

		Global		Bahrain		Kuwait		Qatar		Oman		Saudi Arabia		UAE	
Cause	Sex	YLDs (95% UI)	% Change	YLDs (95% UI)	% Change	YLDs (95% UI)	% Change	YLDs (95% UI)	% Change	YLDs (95% UI)	% Change	YLDs (95% UI)	% Change	YLDs (95% UI)	% Change
Rheumatoid arthritis	Total	29 (20–40)	11.62 (10.28–12.86)	20 (14–28)	34.72 (19.91–52.45)	15 (10–21)	29.30 (12.20–47.58)	14 (9–19)	29.23 (13.27–48.01)	13 (8–17)	38.07 (21.87–57.42)	13 (9–18)	38.85 (21.41–58.75)	12 (8–17)	21.66 (6.23–38.78)
	Men	18 (12–24)	13.73 (11.90–15.55)	10 (7–15)	35.59 (10.97–67.19)	10 (6–14)	20.46 (−1.14–48.50)	10 (7–15)	29.81 (6.03–58.20)	9 (6–12)	37.72 (13.52–66.30)	9 (6–13)	35.22 (12.87–62.34)	9 (6–12)	23.76 (2.53–50.93)
	Women	40 (28–54)	11.13 (9.65–12.50)	38 (26–53)	47.28 (27.02–72.31)	22 (14–30)	27.65 (6.92–53.12)	26 (17–35)	40.43 (17.34–67.29)	20 (13–28)	42.97 (20.07–73.78)	19 (13–27)	41.42 (16.76–70.41)	22 (14–30)	28.66 (7.29–56.34)
Osteoarthritis	Total	228 (115–453)	6.45 (4.84–7.96)	189 (95–376)	6.98 (2.76–11.86)	197 (99–392)	6.34 (2.24–11.00)	189 (95–380)	4.24 (−0.57–9.19)	186 (93–364)	18.09 (13.19–23.32)	233 (119–458)	8.39 (4.24–12.86)	184 (93–363)	7.49 (2.77–12.58)
	Men	187 (94–370)	7.40 (5.83–8.81)	172 (86–341)	6.79 (0.67–13.45)	178 (88–350)	5.60 (−0.31–12.11)	179 (90–363)	5.37 (−0.66–11.44)	167 (84–328)	18.02 (11.30–25.05)	213 (108–412)	7.67 (2.24–13.83)	172 (86–342)	8.71 (2.31–15.46)
	Women	265 (134–523)	6.46 (4.74–8.17)	215 (109–429)	9.76 (4.02–15.50)	224 (112–448)	10.08 (4.29–15.42)	222 (113–435)	5.99 (0.82–11.82)	211 (107–418)	18.79 (12.72–26.01)	264 (136–525)	10.42 (5.67–16.01)	215 (109–425)	10.51 (4.73–16.41)
Osteoarthritis hip	Total	13 (6–26)	12.21 (10.55–14.13)	10 (5–21)	21.04 (9.23–34.86)	11 (5–23)	17.46 (6.91–29.23)	12 (5–24)	18.06 (6.56–32.62)	9 (4–20)	49.54 (35.07–66.69)	10 (5–21)	33.79 (21.21–48.17)	10 (5–21)	28.51 (15.23–43.10)
	Men	12 (6–26)	14.61 (12.77–16.81)	11 (5–22)	16.89 (2.42–33.76)	12 (5–24)	16.48 (1.97–32.26)	12 (6–25)	13.51 (−0.06–30.59)	10 (5–21)	44.42 (26.13–65.05)	11 (5–22)	30.15 (13.72–49.92)	11 (5–22)	24.73 (8.46–42.65)
	Women	13 (6–26)	10.86 (8.89–13.25)	9 (4–19)	23.30 (8.70–41.91)	10 (5–21)	25.72 (9.96–44.83)	10 (5–22)	17.62 (2.89–35.05)	9 (4–19)	52.86 (32.59–75.52)	10 (5–20)	39.07 (21.24–61.46)	9 (4–19)	30.28 (14.40–49.91)
Osteoarthritis knee	Total	138 (68–281)	11.08 (9.94–12.15)	125 (62–256)	8.72 (3.37–14.62)	131 (64–266)	8.00 (2.51–13.98)	126 (63–258)	5.80 (−0.75–12.10)	123 (61–248)	21.07 (14.71–27.89)	126 (62–255)	13.60 (7.33–19.73)	122 (61–248)	9.51 (3.10–15.95)
	Men	112 (55–227)	10.75 (9.42–12.01)	114 (57–232)	7.95 (0.73–16.50)	119 (58–240)	6.86 (−1.51–15.41)	119 (59–245)	6.40 (−2.52–14.58)	111 (55–224)	19.83 (11.24–29.01)	115 (57–233)	12.30 (3.89–21.32)	115 (57–233)	10.09 (1.59–18.78)
	Women	163 (80–331)	11.88 (10.68–13.09)	142 (70–289)	11.98 (3.84–19.59)	148 (73–302)	12.08 (4.37–20.13)	147 (74–298)	7.76 (0.47–16.12)	138 (68–282)	22.70 (14.56–32.35)	143 (71–291)	16.23 (8.01–25.13)	142 (71–288)	13.08 (5.39–21.70)
Osteoarthritis hand	Total	54 (27–111)	−5.67 (−8.35– −3.21)	26 (13–55)	−5.59 (−10.37– −0.36)	27 (13–57)	−1.29 (−6.26–3.98)	23 (11–47)	−11.63 (−17.02– −6.19)	27 (13–58)	−1.15 (−6.19–4.06)	70 (35–144)	−2.99 (−6.68–1.15)	24 (12–49)	−10.03 (−14.98– −4.43)
	Men	38 (19–79)	−4.70 (−7.42– −2.17)	18 (9–37)	−2.00 (−10.13–7.89)	18 (9–37)	−3.21 (−10.81–5.74)	18 (9–36)	−2.36 (−10.44–7.58)	18 (9–37)	0.58 (−7.48–9.89)	58 (29–118)	−3.53 (−8.53–1.70)	18 (9–37)	−3.21 (−11.38–5.92)
	Women	68 (34–140)	−5.39 (−8.26– −2.59)	40 (19–84)	0.58 (−5.29–6.78)	40 (20–83)	0.60 (−5.32–6.90)	39 (20–83)	−2.11 (−7.69–4.06)	40 (20–84)	2.04 (−4.44–9.02)	88 (45–183)	−0.55 (−4.99–4.40)	40 (20–84)	−1.11 (−6.48–5.08)
Osteoarthritis other	Total	23 (11–48)	8.84 (7.48–10.11)	27 (13–57)	8.22 (1.16–15.88)	28 (14–59)	2.80 (−4.22–10.14)	29 (14–60)	7.51 (−0.31–15.95)	26 (13–55)	19.43 (11.80–28.51)	27 (13–58)	10.16 (2.67–18.38)	28 (13–57)	10.07 (2.25–18.08)
	Men	25 (12–53)	10.34 (8.65–11.94)	29 (14–61)	4.72 (−3.95–14.80)	30 (15–63)	2.67 (−5.73–11.90)	31 (15–63)	3.33 (−5.31–13.65)	28 (14–59)	16.31 (6.61–28.35)	29 (14–62)	8.00 (−1.14–18.55)	29 (14–60)	6.33 (−2.55–16.57)
	Women	22 (11–45)	7.24 (5.75–8.63)	24 (12–50)	8.81 (−0.69–20.39)	25 (12–51)	9.54 (−1.05–21.48)	25 (12–52)	5.22 (−3.72–15.50)	24 (12–49)	19.86 (8.30–31.95)	24 (12–51)	13.00 (1.91–24.72)	24 (12–49)	10.63 (0.16–21.69)
Low back pain	Total	780 (549–1,046)	−13.78 (−14.90– −12.82)	809 (569–1,084)	−1.04 (−6.04–3.98)	836 (589–1,121)	−2.97 (−7.04–1.36)	840 (592–1,133)	−0.40 (−5.21–5.02)	813 (568–1,092)	0.97 (−3.76–5.86)	803 (568–1072)	−2.00 (−6.20–2.56)	799 (560–1,068)	−2.22 (−7.47–3.09)
	Men	671 (471–897)	−13.36 (−14.52– −12.26)	826 (579–1,112)	−2.82 (−8.63–3.55)	824 (574–1,110)	−2.27 (−7.97–3.50)	848 (596–1,144)	−2.13 (−7.85–4.40)	826 (575–1,118)	−0.66 (−6.53–5.46)	821 (573–1105)	−3.19 (−9.05–2.55)	816 (570–1,087)	−5.08 (−11.56–1.59)
	Women	884 (625–1,187)	−14.04 (−15.22– −13.03)	773 (540–1,035)	0.26 (−6.01–6.83)	852 (602–1,142)	0.78 (−5.16–6.83)	792 (557–1,066)	−1.44 (−7.11–4.94)	773 (546–1,035)	1.04 (−5.03–7.44)	773 (548–1022)	0.16 (−5.99–6.68)	741 (520–992)	2.24 (−4.27–9.03)
Neck pain	Total	267 (176–384)	2.72 (0.53–4.97)	264 (175–382)	−2.04 (−5.58–1.55)	215 (141–314)	−0.15 (−3.62–3.69)	248 (166–357)	−3.29 (−6.90–0.20)	265 (179–384)	0.44 (−3.16–4.00)	269 (179–389)	−2.22 (−5.43–1.26)	251 (167–364)	−3.84 (−7.46–0.09)
	Men	235 (154–336)	2.71 (0.00–5.44)	219 (144–318)	−1.59 (−6.55–3.16)	176 (116–259)	−3.09 (−8.15–2.29)	219 (143–317)	−2.07 (−6.49–2.48)	220 (144–318)	0.59 (−4.32–5.55)	218 (144–315)	−3.53 (−7.80–0.93)	219 (142–320)	−2.93 (−7.89–2.52)
	Women	299 (197–429	2.74 (−0.08–5.63)	345 (230–510)	0.85 (−3.22–5.14)	266 (176–391)	0.62 (−3.53–5.08)	344 (228–504)	−1.84 (−5.54–2.37)	347 (231–510)	2.09 (−1.84–6.05)	346 (224–505)	−0.77 (−4.61–3.12)	346 (228–508)	−0.96 (−4.59–3.27)
Gout	Total	20 (13–29)	25.94 (23.40–28.94)	19 (12–27)	14.12 (−0.85–31.00)	19 (12–28)	2.18 (−10.05–17.19)	23 (15–33)	16.20 (0.49–35.05)	18 (11–26)	29.06 (11.72–50.19)	19 (12–27)	15.13 (0.72–32.80)	21 (13–30)	17.78 (0.93–37.41)
	Men	32 (20–46)	25.39 (22.72–28.56)	26 (16–37)	6.40 (−8.46–24.70)	26 (17–38)	5.34 (−8.71–23.60)	27 (17–39)	4.18 (−10.89–22.16)	25 (16–36)	22.72 (4.93–44.90)	26 (16–37)	12.15 (–4.40–31.44)	25 (16–37)	8.67 (−8.15–28.48)
	Women	9 (6–13)	24.25 (21.44–27.58)	8 (5–12)	11.90 (−11.45–37.20)	9 (5–13)	12.53 (−8.00–39.36)	8 (5–12)	6.58 (−14.26–34.35)	8 (5–11)	25.87 (0.03–58.28)	8 (5–12)	17.87 (–5.75–48.46)	8 (5–11)	12.49 (−9.66–41.15)
Other MSK disorders	Total	466 (318–648)	36.23 (32.48–40.80)	433 (298–598)	34.90 (24.95–45.79)	483 (327–672)	15.15 (1.40–29.74)	408 (274–567)	48.66 (36.07–63.13)	416 (282–587)	78.77 (61.01–99.39)	379 (253–527)	92.73 (71.45–120.92)	415 (282–579)	43.74 (31.15–57.85)
	Men	362 (244–508)	44.15 (38.69–51.34)	363 (250–511)	42.90 (26.10–61.38)	381 (250–538)	14.36 (–2.54–33.88)	358 (237–502)	72.18 (50.13–102.25)	344 (232–489)	105.63 (74.41–147.64)	278 (180–394)	137.77 (90.51–220.43)	361 (243–506)	51.71 (34.03–74.21)
	Women	566 (390–784)	31.69 (28.56–35.27)	551 (376–760)	31.19 (20.47–43.33)	613 (421–854)	13.27 (0.40–28.57)	579 (394–805)	31.31 (20.28–43.78)	548 (369–761)	61.29 (45.37–80.27)	531 (360–737	67.53 (49.94–87.99)	571 (391–796)	39.48 (26.63–53.42)

GCC, Gulf Cooperation Council; MSK, musculoskeletal; UI, uncertainty interval; YLDs, years lived with disability; % Change, percentage change between 1990 and 2019.

The specific MSK disorders that have shown the largest increases in the age-standardized YLDs per 100,000 individuals among all the GCC countries between 1990 and 2019 were rheumatoid arthritis and osteoarthritis hip (Table 4). The largest increase in age-standardized YLDs per 100,000 individuals with rheumatoid arthritis was observed in Saudi Arabia [38.85% (21.41–58.75)], and the smallest increase was observed in UAE [21.66% (6.23–38.78)]. The largest increase in age-standardized YLDs per 100,000 individuals with osteoarthritis hip was observed in Oman [49.54% (35.07–66.69)], and the smallest increase was observed in Kuwait [17.46% (6.91–29.23)].

### Attributable burden

In 2019, across both sexes and all ages, 19.50% (95% UI: 16.51–22.67) of global YLDs resulting from MSK disorders were attributable to exposure to four GBD risk factors (Supplementary Appendix Table 2). Specifically, 10.35% (12.01–8.80) of YLDs were attributable to occupational ergonomic factors, 6.85% (5.07–8.97) to smoking, 4.98% (7.55–2.91) to high BMI, and 0.16% (0.11–0.22) to kidney dysfunction. In GCC countries, high BMI had the highest contribution to YLDs from MSK disorders in Kuwait [10.50% (6.98–14.17)], Saudi Arabia [10.01% (13.49–6.66)], Bahrain [9.01% (5.78–12.44)], and Oman [8.95% (5.78–12.40)]. Whereas in Qatar and UAE, the exposure to occupational ergonomic factors had the highest contribution to YLDs from MSK disorders [11.79% (10.2–13.69) and 11.22% (9.62–13.09), respectively].

## Discussion

The main aim of this study was to evaluate the prevalence of MSK disorders in the GCC countries and YLDs between 1990 and 2019 using the findings on MSK disorders from the GBD 2019. This study also aimed to assess the attributable burden from the risk factors of MSK disorders.

In terms of prevalence, out of all the diseases studied, MSK disorders were the fifth most common in Kuwait, sixth in Bahrain, Oman, Qatar, and UAE, and seventh in Saudi Arabia. Age-standardized prevalence of MSK disorders was highest in Kuwait and lowest in Oman. Age-standardized prevalence increased in all GCC countries between 1990 and 2019, with the largest increase being reported by Oman. The most prevalent MSK disorder globally was LBP, which in the GCC was most commonly reported in Qatar. However, analyses of the findings of the 2019 study indicate that the two MSK disorders which have shown the largest increase in age standardization prevalence among all the GCC countries between 1990 and 2019 are rheumatoid arthritis and osteoarthritis hip. The largest increase in age-standardized prevalence of rheumatoid arthritis was observed in Saudi Arabia, and the smallest increase was observed in UAE. The largest increase in age-standardized prevalence of osteoarthritis hip was observed in Oman, and the smallest increase was observed in Qatar.

Between 1990 and 2019, MSK disorders were the second leading cause of YLDs. The highest age-standardized YLDs per 100,000 individuals with MSK disorders in 2019 for GCC countries was Kuwait. An increase in age-standardized YLDs of MSK disorders between 1990 and 2019 was found in all GCC countries with the largest increase occurring in Oman. The largest increase in age-standardized YLDs per 100,000 individuals among GCC countries in this period was in rheumatoid arthritis and osteoarthritis hip. The largest increase in rheumatoid arthritis in GCC countries was in Saudi Arabia, while the largest increase in osteoarthritis hip was in Oman. Finally, it was found that high BMI was the greatest contributor to attributable burden of MSK disorders in Saudi Arabia, Bahrain, and Oman, but in Qatar and UAE, the majority of MSK disorders were attributable to occupational ergonomic factors.

The GCC countries are classified as high-income countries (22). As such, the results of the GBD Survey for MSK disorders among GCC countries can be compared to those for Canada or the United Kingdom, to extract useful public policy messages. Prevalence of MSK disorders ranks fifth to seventh among the most common diseases in the GCC countries. By the way of comparison, MSK disorders rank as the third most common disease among the Canadian population (23). In the GCC, the most common MSK disorders are rheumatoid arthritis and osteoarthritis hip. However, in Canada, the most common MSK condition is LBP followed by osteoarthritis knee and hip (23). Significantly, Canada is among the top 10 countries in the world for the prevalence of the latter condition (23). In the United Kingdom, the two most common MSK disorders are back pain and osteoarthritis (24). These examples indicate that the GCC countries have a lower prevalence of LBP compared to some high-income countries and higher levels of rheumatoid arthritis. In terms of YLDs, MSK disorders are the leading cause of YLDs in the United Kingdom increasing from 1,322 per 100,000 of the population in 1990 to 1,392 per 100,000 of the population in 2017 (24). During the same period, age prevalence for all MSK disorders has increased in all the GCC countries, with the highest rise occurring in Oman. Similarly, age prevalence of MSK disorders in Canada increased from 23% in 1990 to 27.8% in 2017 (23). These examples indicate that some high-income countries report similar trends relating to YLDs and MSK disorders.

The data found that the prevalence of YLDs from MSK disorders was highest in Kuwait and Saudi Arabia. In Kuwait, both lifestyle factors and lack of awareness of risk factors in the workplace may contribute to YLDs from MSK disorders. Akrouf and Crawford (25) looked at the pattern of MSK suffered by bank office workers in Kuwait. They found that 80% of the 750 employees surveyed had suffered at least one episode of MSK during the past year and 42% had experienced a disabling episode of MSK (25). Significantly, they found that smoking, drinking alcohol, gender, and marital status all contributed to the development of MSK disorders in this group. Similarly, Kirsch Micheletti and Bláfoss (26) found that smoking but not the consumption of alcohol was associated with a higher risk of MSK pain. It may also be the case that lack of education about the risk factors in the workplace may contribute to higher YLDs of MSK disorders in Kuwait as Alnaser and Aljadi (27) found that prevalence of work activities was the highest risk factor associated with developing an MSK disorder. According to the 2010 and 2017 GBD surveys, MSK disorders were the second leading cause of DALYs in Saudi Arabia (10). These disorders are particularly prevalent in older adults of the Saudi Arabian population (10). It has been found that high BMI is a major risk factor for disease burden among the Saudi Arabian population (28). These findings, therefore, suggest both lifestyle factors and lack of awareness of MSK disorders contribute to a high prevalence of YLDs of these disorders in Saudi Arabia and Kuwait.

While the prevalence of YLDs was the lowest in Oman, the results of the GBD study indicate that the YLDs of MSK disorders are sharply increasing. There is not much literature available on this topic, but what is indicated that the potential reasons for the low prevalence of MSK disorders and the increase of YLDs in the Omani population include lack of awareness of MSK disorders in the past, lack of understanding concerning how MSK disorders can be treated, high levels of joint laxity in the Omani population, and high BMI (29, 30). The study conducted by Pountain Gillian (29) in Oman found that 42% of female subjects reported back pain as did 25% of male subjects, while 15% of women and 18% of men reported knee pain (29). Notably though, only one man (0.2%) and three women (0.6%) reported hip pain; and MSK pain was more common in rural communities. The study also found that joint mobility and laxity scores were higher in women than men, and these scores declined with aging. As well as in this, it was found that BMI was higher in women who reported knee or back pain than in those without these pains; but in men, only knee pain was associated with a higher BMI (29). Furthermore, another study found that over 50% of the Omani population are overweight or obese, with 30% of the population having a BMI of over 30; and obesity rates have increased considerably in women since 2008 (31). Therefore, it may be the case that increased rates of obesity may be contributing to an increase in the YLDs of MSK disorders in Oman.

There is some indication that there is a lack of awareness concerning MSK disorders and how these conditions can be treated in the medical community in Oman. This may account for the low rates of prevalence of MSK disorders in Oman until recently and for the increase in diagnosis in such conditions recently. For example, to remedy the lack of awareness of MSK disorders, the Omani Ministry of Health recently organized a first-of-its-kind scientific conference on the rehabilitation of lower limbs in MSK disorders in July 2021 (32). This conference looked at how these disorders can be diagnosed and the therapeutic and rehabilitation methods that can be used to treat these disorders (32).

The analysis of these data suggests that although Oman has the least prevalence of MSK disorders and YLDs compared to other GCC, Oman has the sharpest increase in prevalence and YLDs rate, suggesting that metabolic, behavioral, and environmental or occupational risk factors are increasing in the area. Additionally, Oman’s life expectancy has increased considerably in the last four decades, rising from 50 years in 1970 to 74.22 years in 2011 (33). The sociodemographic patterns in Oman are said to be in an “epidemiological transition” phase, characterized by a “shift away from acute infectious and deficiency diseases associated with underdevelopment and toward chronic NCDs associated with modernization and advanced levels of development.” (34). That requires a call for preventative action by integrating MSK disease preventive and control programs in national health programs through public education, occupational health and safety, and ergonomics are critical components of any preventive and control program. In addition, medical treatments and rehabilitation to maintain functional status are crucial for situational control.

Our findings indicate a significant prevalence of osteoarthritis and rheumatoid arthritis in Saudi Arabia and the UAE. A newly published systematic review of the prevalence of osteoarthritis in the GCC found an overall prevalence of 16.6 across all GCC nations (35). However, it should be highlighted that more than half of the research reviewed in the systematic review was from Saudi Arabia, with no publications from Bahrain or Kuwait. As a result, there may be an under-reported prevalence in certain GCC nations. Similarly, Almoallim, Al Saleh (36) highlighted significant heterogeneity in the prevalence and incidence of rheumatoid arthritis in various GCC countries due to the lack of data registry and research examining the epidemiology of rheumatoid arthritis.

Musculoskeletal disorders were attributable to exposure to four GBD risk factors: occupational ergonomic factors, smoking, high BMI, and kidney dysfunction. According to the World Health Organization, the GCC nations have the highest incidence of obesity. Kuwait, Bahrain, Saudi Arabia, and the UAE are among the top 10 obese nations globally (11). A previous study, conducted among construction workers in GCC countries, found that approximately 47% of workers experienced MSK pain, and a majority of them were overweight or obese (37). Obesity is also considered a risk factor for hypertension which is also a risk factor for kidney dysfunction (38). In addition, the burden of MSK pain is projected to rise significantly in the GCC countries due to population growth, aging, and the emergence of new risk factors for MSK diseases such as obesity, injury, and a sedentary lifestyle.

Recognizing the impact of MSK disorders on other health diseases is important. For instance, MSK pain leads to mobility limitation and disability, especially in older people, which results in loss of independence, frailty, cognitive decline and sarcopenia, and the risk of NCDs (i.e., those associated with premature mortality such as diabetes, heart disease, and cancer). Furthermore, MSK pain should be recognized as one of the major reasons for the lack of adherence to rehabilitation programs and healthy behaviors. A framework to initiate and develop a high-value care model for MSK disorders in GCC countries is important, such as the “Australian National Pain Strategy” and “Relieving Pain in America National Blueprint”, developed in Australia and the United States, respectively (39–41). Future research is recommended to have a better estimate of MSK disorders in GCC countries; and a dual strategy is needed to fortify the evidence base and a policy response which include the integration of MSK disorders into health surveillance systems for injury and NCDs.

The lack of primary data is a significant drawback of the GBD study of the burden of illnesses and injuries. When information is not available, the results are based on the out-of-sample predictive validity of the modeling attempts. While improvements in data processing and modeling may result in small gains in prediction accuracy, more and better primary data collection is needed for significant changes. Even if data exist, it is likely it was not gathered using the best-case definition or measurement method. The more precise identification of the preferred and alternative measurement techniques for each outcome and the bias mapping from alternative to reference method conducted as part of GBD 2019 have resulted in more consistency in data modifications (16). These improvements will also aid in the establishment of data collection priorities, preferred case definitions, and research methods.

Moreover, the lack of high-quality data in the area may affect the predicted MSK disease trend. Although this issue arises in various diseases, it may have a disproportionate effect on MSK problems compared to other conditions. Therefore, these limitations should be considered a research opportunity within the GCC countries and call for a data registry for some GCC countries to monitor and prevent the MSK-related burden.

In conclusion, this study demonstrated an increase in both age-standardized prevalence of MSK disorders and YLDs between 1990 and 2019 which was observed for all GCC countries. Furthermore, risk factors such as higher BMI and exposure to occupational ergonomic factors were highly associated with YLDs due to MSK disorders. The results of this study provide guidance for the potential nature of preventative and management programs to optimize individuals’ health.

## Data availability statement

All the data and analyzes used in this study were obtained from the GBD study 2019, which is publicly available online on the website of the Institute of Health Metrics and Evaluation (https://vizhub.healthdata.org/gbd-compare/).

## Author contributions

HA, ST, and YZ: substantial contribution to the conception and design of the study. HA and MAA: substantial contributions to the acquisition and substantial contributions to analysis or interpretation of data. HA, MAA, MA, AA, FA, and DA: draft the work. All authors revised the work critically for important intellectual content and provided approval for publication of the content, contributed to the article, and approved the submitted version.

## Footnotes

1
https://vizhub.healthdata.org/gbd-compare/

2
https://www.gcc-sg.org/en-us/Pages/default.aspx
