# Corrigendum: Burden of musculoskeletal disorders in the gulf cooperation council countries, 1990–2019: findings from the global burden of disease study 2019

**DOI:** 10.3389/fmed.2023.1249504

**Published:** 2023-07-13

**Authors:** Hosam Alzahrani, Mansour A. Alshehri, Mazyad Alotaibi, Ahmed Alhowimel, Faris Alodaibi, Dalyah Alamam, Yan Zheng, Stefanos Tyrovolas

**Affiliations:** ^1^Department of Physical Therapy, College of Applied Medical Sciences, Taif University, Taif, Saudi Arabia; ^2^Physiotherapy Department, Faculty of Applied Medical Sciences, Umm Al-Qura University, Mecca, Saudi Arabia; ^3^NHMRC Centre of Clinical Research Excellence in Spinal Pain, Injury and Health, School of Health and Rehabilitation Sciences, University of Queensland, Brisbane, QLD, Australia; ^4^Department of Health and Rehabilitation Sciences, College of Applied Medical Sciences, Prince Sattam Bin Abdulaziz University, Al-Kharj, Saudi Arabia; ^5^Department of Health Rehabilitation Sciences, King Saud University, Riyadh, Saudi Arabia; ^6^WHO Collaborating Centre for Community Health Services (WHOCC), School of Nursing, The Hong Kong Polytechnic University, Hong Kong, Hong Kong SAR, China; ^7^Research, Innovation and Teaching Unit, Parc Sanitari Sant Joan de Déu, Fundacio Sant Joan de Deu, Sant Boi de Llobregat, Spain; ^8^Instituto de Salud Carlos III, Centro de Investigación Biomédica en Red de Salud Mental (CIBERSAM), Madrid, Spain

**Keywords:** global burden of disease, Gulf Cooperation Council (GCC), musculoskeletal – disorders, prevalence, years lived with disability

In the published article, there was an error. There was an error in the Abstract.

A correction has been made to **Abstract, Results section**. This section previously stated: **Results:** MSK disorders prevalence ranked fifth in Kuwait, sixth in Bahrain, Oman, Qatar, and UAE, and seventh in Saudi Arabia among all the diseases in 2019. For all GCC countries, MSK disorders were ranked the second leading cause of disability as measured by YLDs for the years 1990 and 2019. The age-standardized prevalence of MSK disorders in 2019 for Bahrain, Kuwait, Oman, Qatar, Saudi Arabia, and UAE was 18.56% (95% UI: 17.51–19.66), 19.35% (18.25–20.52), 18.23% (17.14–19.36), 18.93% (17.81–20.06), 19.05% (17.96–20.22), and 18.26% (17.18–19.38), respectively. The age-standardized YLDs per 100,000 individuals of MSK disorders in 2019 for Bahrain, Kuwait, Oman, Qatar, Saudi Arabia, and UAE were 1,734 (1,250–2,285), 1,764 (1,272–2,322), 1,710 (1,224–2,256), 1,721 (1,246–2,274), 1,681 (1,207–2,235), and 1,715 (1,230–2,274), respectively. For risk factors, high body mass index (BMI) had the highest contribution to MSK disorders YLDs in most GCC countries (Bahrain, Kuwait, Oman, and Saudi Arabia), followed by the exposure to occupational ergonomic factors which had the highest contribution to MSK disorders YLDs in Qatar and UAE.

The corrected section appears below:

**Results:** MSK disorders prevalence ranked fifth in Kuwait, sixth in Bahrain, Oman, Qatar, and UAE, and seventh in Saudi Arabia among all the diseases in 2019. For all GCC countries, MSK disorders were ranked the second leading cause of disability as measured by YLDs for the years 1990 and 2019. The age-standardized prevalence of MSK disorders in 2019 for Bahrain, Kuwait, Oman, Qatar, Saudi Arabia, and UAE was 18.56% (95% UI: 17.51–19.66), 19.35% (18.25–20.52), 18.23% (17.14–19.36), 18.93% (17.81–20.06), 19.05% (17.96–20.22), and 18.26% (17.18–19.38), respectively. The age-standardized YLDs per 100,000 individuals of MSK disorders in 2019 for Bahrain, Kuwait, Oman, Qatar, Saudi Arabia, and UAE were 1,734 (1,250–2,285), 1,764 (1,272–2,322), 1,710 (1,224–2,256), 1,721 (1,246–2,274), 1,715 (1,230–2,274), and 1,681 (1,207–2,235), respectively. For risk factors, high body mass index (BMI) had the highest contribution to MSK disorders YLDs in most GCC countries (Bahrain, Kuwait, Oman, and Saudi Arabia), followed by the exposure to occupational ergonomic factors which had the highest contribution to MSK disorders YLDs in Qatar and UAE.

The authors apologize for this error and state that this does not change the scientific conclusions of the article in any way. The original article has been updated.

