# Supplementary material for: Burden of musculoskeletal disorders in the gulf cooperation council countries, 1990–2019: Findings from the global burden of disease study 2019
Source: Front Med (Lausanne). 2022 Oct 4;9:855414. doi: 10.3389/fmed.2022.855414 (PMC9577605; doi:10.3389/fmed.2022.855414)
Supplement: Supplementary file 1 [file Data_Sheet_1.PDF]

Appendix Table 1. List of International Classification of Diseases (ICD) codes mapped to the Global Burden of Disease cause list

Cause (GBD name)	ICD10	ICD10 Used in Hospital/Claims Analyses	ICD9	ICD9 Used in Hospital/Claims Analyses
**MSK disorders (all)**	G54.2-G54.4, I27.1, L93-L93.2, M00-M03.6, M05-M10.19, M10.3-M25.9, M30-M32.9, M34-M36.8, M40-M43.9, M45-M49, M49.2-M51.9, M53-M54.9, M61-M63.89, M65-M68.8, M70-M72.4, M72.8-M73, M75-M77.9, M79-M79.676, M79.8-M87.09, M87.2-M89.59, M89.7-M95.9, M99-M99.9, Z13.82-Z13.83, Z82.6-Z82.69, Z87.3-Z87.39	G54.2-G54.4, I27.1, L93-L93.2, M00-M10.19, M10.3-M25.9, M30-M48.58, M49.81-M54.5, M61-M72.4, M72.8-M87.09, M87.2-M95.9, M99-M99.9	274-274.9, 353.1-353.4, 416.1, 446-446.9, 710-710.2, 710.5-724.9, 726-727.9, 729-730.39, 730.7-739.9, V13.4-V13.59, V17.7-V17.89, V43.6-V43.8, V58.64-V58.65, V58.78, V77.5, V82.1-V82.2	274-274.9, 353.1-353.4, 416.1, 446-446.9, 710-727.9, 729-730.39, 730.7-739.9
**Rheumatoid arthritis**	M05-M05.9, M08-M09.8	M05-M05.9	714-714.9	714-714.9
**Osteoarthritis**	M16-M18.9	M16-M19.93	715-715.98	715-715.98, 731-731.9
**Osteoarthritis hip**	M16-M16.9		715.15, 715.25, 715.35	
**Osteoarthritis knee**	M17-M17.9			
**Osteoarthritis hand**	M18-M18.9		715.11-715.14, 715.16-715.17, 715.21-715.24, 715.26-715.27, 715.30-715.34, 715.36-715.37	
**Osteoarthritis other**			715-715.10, 715.18-715.20, 715.28-715.3, 715.38-715.98	

**Low back pain**	G54.4, M47.015-M47.019, M47.15-M47.18, M47.25-M47.28, M47.815-M47.818, M47.896-M47.899, M48.05-M48.08, M48.16-M48.19, M48.25-M48.27, M48.35-M48.38, M48.45-M48.48, M48.55-M48.58, M49.85-M49.88, M51.05-M51.07, M51.15-M51.17, M51.25-M51.27, M51.35-M51.37, M51.45-M51.47, M51.85-M51.87, M53.3, M53.85-M53.88, M54.05-M54.09, M54.15-M54.18, M54.3-M54.5, M99.03-M99.04, M99.13-M99.14, M99.23-M99.24, M99.33-M99.34, M99.43-M99.44, M99.53-M99.54, M99.63-M99.64, M99.73-M99.74, M99.83-M99.84	G54.4, M47.015-M47.019, M47.15-M47.18, M47.25-M47.28, M47.815-M47.818, M47.896-M47.899, M48.05-M48.08, M48.16-M48.19, M48.25-M48.27, M48.35-M48.38, M48.45-M48.48, M48.55-M48.58, M49.85-M49.88, M51.05-M51.87, M53.3, M53.85-M53.88, M54.05-M54.09, M54.15-M54.18, M54.3-M54.5, M99.03-M99.04, M99.13-M99.14, M99.23-M99.24, M99.33-M99.34, M99.43-M99.44, M99.53-M99.54, M99.63-M99.64, M99.73-M99.74, M99.83-M99.84	353.1, 353.4, 721.3, 721.42, 722.10, 722.32, 722.52, 722.73, 722.83, 722.93, 724.02-724.03, 724.2-724.3, 724.6-724.79	353.1, 353.4, 721.3-721.42, 722.10-722.52, 722.73, 722.83, 722.93-724.79
**Neck pain**	G54.2, M47.011-M47.013, M47.11-M47.13, M47.21-M47.23, M47.811-M47.813, M47.892-M47.894, M48.01-M48.03, M48.12-M48.14, M48.21-M48.23, M48.31-M48.33, M48.41-M48.43, M48.51-M48.53, M49.81-M49.83, M50-M50.93, M53.0-M53.1, M53.81-M53.83, M54.01-M54.03, M54.11-M54.13, M54.2, M54.81, M99.01, M99.11, M99.21, M99.31, M99.41, M99.51, M99.61, M99.71, M99.81	G54.2, M47.011-M47.013, M47.11-M47.13, M47.21-M47.23, M47.811-M47.813, M47.892-M47.894, M48.01-M48.03, M48.12-M48.14, M48.21-M48.23, M48.31-M48.33, M48.41-M48.43, M48.51-M48.53, M49.81-M49.83, M50-M50.93, M53.0-M53.1, M53.81-M53.83, M54.01-M54.03, M54.11-M54.13, M54.2, M99.01, M99.11, M99.21, M99.31, M99.41, M99.51, M99.61, M99.71, M99.81	353.2, 721.0-721.1, 722.0, 722.71, 722.81, 722.91, 723-723.9	353.2, 721.0-721.1, 722.0, 722.71, 722.81, 722.91

**Gout**	M10-M10.19, M10.3-M10.9, M1A00X0-M1A9XX1	M10-M10.19, M10.3-M10.9, M1A.00X.0-M1A.9XX.1	274-274.9, 712.0-712.09	274-274.9, 712.0-712.09
**Other MSK disorders**	G54.3, I27.1, L93-L93.2, M00-M03.6, M06-M07.69, M11-M15.9, M19-M19.93, M20-M25.9, M30-M32.9, M34-M36.8, M40-M43.9, M45-M47.01, M47.014, M47.02-M47.10, M47.14, M47.2-M47.20, M47.24, M47.8-M47.81, M47.814, M47.819-M47.891, M47.895, M47.9-M48.00, M48.04, M48.1-M48.11, M48.15, M48.2-M48.20, M48.24, M48.3-M48.30, M48.34, M48.4-M48.40, M48.44, M48.5-M48.50, M48.54, M48.8-M49, M49.2-M49.80, M49.84, M49.89, M51-M51.04, M51.1-M51.14, M51.2-M51.24, M51.3-M51.34, M51.4-M51.44, M51.8-M51.84, M51.9, M53, M53.2, M53.8-M53.80, M53.84, M53.9-M54.00, M54.04, M54.1-M54.10, M54.14, M54.6-M54.8, M54.89-M54.9, M61-M63.89, M65-M68.8, M70-M72.4, M72.8-M73, M75-M77.9, M79-M79.676, M79.8-M87.09, M87.2-M89.59, M89.7-M95.9, M99-M99.00, M99.02, M99.05-M99.10, M99.12, M99.15-M99.20, M99.22, M99.25-M99.30, M99.32, M99.35-M99.40, M99.42, M99.45-M99.50, M99.52, M99.55-M99.60, M99.62, M99.65-M99.70, M99.72, M99.75-M99.80, M99.82, M99.85-M99.9	I27.1, L93-L93.2, M00-M03.6, M06-M09.8, M11-M15.9, M20-M25.9, M30-M46.99, M61-M72.4, M72.8-M87.09, M87.2-M95.9, M99, M99.05-M99.09, M99.15-M99.19, M99.25-M99.29, M99.35-M99.39, M99.45-M99.49, M99.55-M99.59, M99.65-M99.69, M99.75-M99.79, M99.85-M99.9	353.3, 416.1, 446-446.9, 710-710.2, 710.5-712, 712.1-713.8, 716-721, 721.2, 721.4-721.41, 721.5-722, 722.1, 722.11-722.31, 722.39-722.51, 722.6-722.70, 722.72, 722.8-722.80, 722.82, 722.9-722.90, 722.92, 724-724.01, 724.09-724.1, 724.4-724.5, 724.8-724.9, 726-727.9, 729-730.39, 730.7-739.9	416.1, 446-446.9, 710-712, 712.1-713.8, 716-720.9, 726-727.9, 729-730.39, 730.7-730.99, 732-739.9

Abbreviations: MSK, musculoskeletal.

Appendix Table 2. The proportion of MSK disorders YLDs attributable to behavioral, metabolic and environmental/occupational risk factors, by cause, in 2019

	**Global**	**Bahrain**	**Kuwait**	**Qatar**	**Oman**	**Saudi Arabia**	**UAE**
**Risk**	**Prevalence (95% UI)**	**Prevalence (95% UI)**	**Prevalence (95% UI)**	**Prevalence (95% UI)**	**Prevalence (95% UI)**	**Prevalence (95% UI)**	**Prevalence (95% UI)**
**All risk factors**	19.50 (22.67-16.51)	20.98 (24.79-17.14)	23.45 (27.51-19.60)	26.01 (30.23-21.93)	19.94 (23.77-16.52)	21.83 (25.47-18.11)	25.16 (29.18-21.34)
**Behavioral risks**	6.85 (8.97-5.07)	6.70 (9.58-4.36)	7.84 (10.65-5.36)	7.47 (10.19-5.11)	4.86 (6.69-3.32)	6.31 (8.86-4.20)	7.81 (10.36-5.66)
Smoking	6.85 (8.97-5.07)	6.70 (9.58-4.36)	7.84 (10.65-5.36)	7.47 (10.19-5.11)	4.86 (6.69-3.32)	6.31 (8.86-4.20)	7.81 (10.36-5.66)
**Metabolic risks**	5.09 (7.62-3.03)	9.15 (12.53-5.93)	10.62 (14.27-7.13)	11.50 (15.35-7.95)	9.06 (12.46-5.91)	10.15 (13.61-6.80)	10.98 (14.59-7.54)
High body-mass index	4.98 (7.55-2.91)	9.01 (12.44-5.78)	10.50 (14.17-6.98)	11.36 (15.23-7.77)	8.95 (12.40-5.78)	10.01 (13.49-6.66)	10.85 (14.48-7.35)
Kidney dysfunction	0.16 (0.22-0.11)	0.27 (0.38-0.18)	0.26 (0.37-0.18)	0.33 (0.48-0.23)	0.23 (0.33-0.16)	0.31 (0.43-0.21)	0.32 (0.44-0.22)
**Environmental/occupational risks**	10.35 (12.01-8.80)	8.30 (9.61-6.97)	9.23 (10.80-7.91)	11.79 (13.69-10.21)	8.79 (10.43-7.29)	9.22 (10.84-7.75)	11.22 (13.09-9.62)
Occupational ergonomic factors	10.35 (12.01-8.80)	8.30 (9.61-6.97)	9.23 (10.80-7.91)	11.79 (13.69-10.21)	8.79 (10.43-7.29)	9.22 (10.84-7.75)	11.22 (13.09-9.62)

Abbreviations: MSK, musculoskeletal; UI, uncertainty interval; YLDs, years lived with disability.
